# Broad and potent activity against SARS-like viruses by an engineered human monoclonal antibody

**DOI:** 10.1126/science.abf4830

**Published:** 2021-01-25

**Authors:** C. Garrett Rappazzo, Longping V. Tse, Chengzi I. Kaku, Daniel Wrapp, Mrunal Sakharkar, Deli Huang, Laura M. Deveau, Thomas J. Yockachonis, Andrew S. Herbert, Michael B. Battles, Cecilia M. O’Brien, Michael E. Brown, James C. Geoghegan, Jonathan Belk, Linghang Peng, Linlin Yang, Yixuan Hou, Trevor D. Scobey, Dennis R. Burton, David Nemazee, John M. Dye, James E. Voss, Bronwyn M. Gunn, Jason S. McLellan, Ralph S. Baric, Lisa E. Gralinski, Laura M. Walker

**Affiliations:** 1 Adimab, LLC, Lebanon, NH 03766, USA.; 2 Department of Epidemiology, The University of North Carolina at Chapel Hill, Chapel Hill, NC 27599, USA.; 3 Department of Molecular Biosciences, The University of Texas at Austin, Austin, TX 78712, USA.; 4 Department of Immunology and Microbiology, The Scripps Research Institute, La Jolla, CA 92037, USA.; 5 Paul G. Allen School of Global Animal Health, Washington State University, Pullman, WA 99164, USA.; 6 U.S. Army Medical Research Institute of Infectious Diseases, Frederick, MD 21702, USA.; 7 The Geneva Foundation, Tacoma, WA 98402, USA.; 8 IAVI Neutralizing Antibody Center, The Scripps Research Institute, La Jolla, CA 92037, USA.; 9 Consortium for HIV/AIDS Vaccine Development (CHAVD), The Scripps Research Institute, La Jolla, CA 92037, USA.; 10 Ragon Institute of Massachusetts General Hospital, Massachusetts Institute of Technology, and Harvard, Cambridge, MA 02139, USA.; 11 Departments of Microbiology and Immunology, The University of North Carolina at Chapel Hill, Chapel Hill, NC 27599, USA.; 12 Adagio Therapeutics, Inc., Waltham, MA 02451, USA.

## Abstract

The recurrent zoonotic spillover of coronaviruses (CoVs) into the human population underscores the need for broadly active countermeasures. We employed a directed evolution approach to engineer three severe acute respiratory syndrome coronavirus 2 (SARS-CoV-2) antibodies for enhanced neutralization breadth and potency. One of the affinity-matured variants, ADG-2, displays strong binding activity to a large panel of sarbecovirus receptor binding domains and neutralizes representative epidemic sarbecoviruses with high potency. Structural and biochemical studies demonstrate that ADG-2 employs a distinct angle of approach to recognize a highly conserved epitope that overlaps the receptor binding site. In immunocompetent mouse models of SARS and COVID-19, prophylactic administration of ADG-2 provided complete protection against respiratory burden, viral replication in the lungs, and lung pathology. Altogether, ADG-2 represents a promising broad-spectrum therapeutic candidate against clade 1 sarbecoviruses.

## Targeting sarbecoviruses

As we continue to battle the COVID-19 pandemic, we must confront the possibility of new pathogenic coronaviruses emerging in humans in the future. With this in mind, Rappazzo *et al.* isolated antibodies from a survivor of the 2003 severe acute respiratory syndrome coronavirus (SARS-CoV), used yeast display libraries to introduce diversity into these antibodies, and then screened for binding to SARS-CoV-2. One of the affinity-matured progeny strongly neutralized SARS-CoV-2, SARS-CoV, and two SARS-related viruses from bats. In addition, this antibody bound to the receptor-binding domains from a panel of sarbecoviruses, suggesting broader activity, and provided protection against SARS-CoV and SARS-CoV-2 in mouse models.

*Science*, this issue p. 823

## Teaser

An affinity-optimized human monoclonal antibody displays broad in vivo efficacy in murine models of SARS and COVID-19.

The COVID-19 pandemic, caused by the β-coronavirus severe acute respiratory syndrome coronavirus 2 (SARS-CoV-2), presents an urgent global health crisis. Although two vaccines and two monoclonal antibody (mAb) therapies have been authorized for emergency use by the U.S. Food and Drug Administration, it is unknown whether these vaccines and treatments will provide broad protection against newly emerging SARS-CoV-2 strains that originate in humans or animal reservoirs (*1*). Furthermore, the recurrent zoonotic spillover of CoVs into the human population, along with the broad diversity of SARS-like CoVs circulating in animal reservoirs (*2*), suggests that new pathogenic CoVs are likely to emerge in the future and underscores the need for broadly active countermeasures.

As in other CoVs, the SARS-CoV-2 spike (S) protein mediates viral entry and is the only known target for neutralizing antibodies (nAbs). Although SARS-CoV and SARS-CoV-2 share 76% amino acid identity in their S proteins, only a limited number of cross-neutralizing antibodies have been described to date (*3*–*6*). These rare broadly neutralizing antibodies (bnAbs) represent an attractive opportunity for therapeutic drug stockpiling to prevent or mitigate future outbreaks of SARS-related CoVs, but their limited neutralization potency may translate into suboptimal protective efficacy or impractical dosing regimens. In this study, we show that such bnAbs can be engineered for improved neutralization potency while retaining neutralization breadth, and we demonstrate that these bnAbs can provide broad protection in vivo.

## Affinity optimization of SARS-CoV-2 antibodies

We isolated several antibodies from the memory B cells of a 2003 SARS survivor that cross-neutralize multiple SARS-related viruses with relatively modest potency (*3*). Although breadth and potency are often opposing characteristics, we sought to engineer these bnAbs for improved neutralization potency against SARS-CoV-2 while maintaining or improving neutralization breadth and potency against other SARS-like viruses. Because binding affinity and neutralization potency are generally well correlated (*7*), we employed yeast surface display technology to improve the binding affinities of three of the bnAbs (ADI-55688, ADI-55689, and ADI-56046) for a prefusion-stabilized SARS-CoV-2 S protein (*3*, *8*–*10*).

**Fig. 1:**
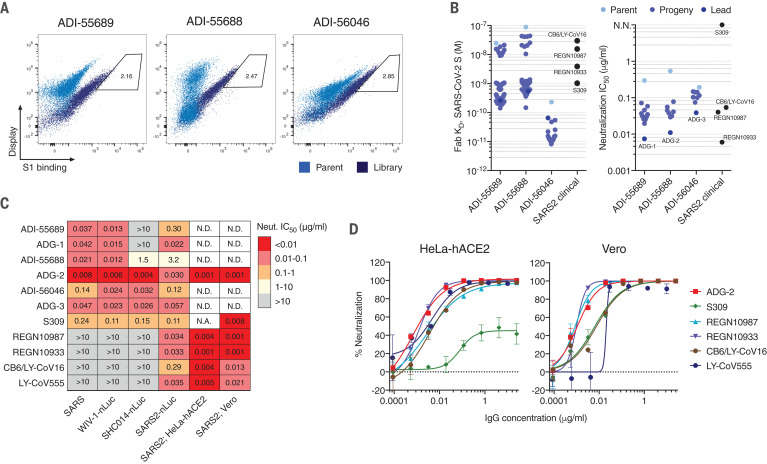
Engineering SARS-CoV-2 antibodies for enhanced neutralization breadth and potency. (**A**) Flow cytometry plots from the terminal round of selection, showing binding of parental antibodies (light blue) and affinity maturation library antibodies (dark blue) to the SARS-CoV-2 S1 protein at 1 nM. Gates indicate the yeast populations sorted for antibody sequencing and characterization. (**B**) Dot plots of Fab binding affinities (left) and MLV–SARS-CoV-2 pseudovirus neutralization IC_50_s (right) of parental antibodies and affinity-matured progeny. Clinical-stage SARS-CoV-2 antibodies are shown for comparison. (**C**) Heatmap showing the neutralization IC_50_s of the indicated antibodies against authentic SARS-CoV, WIV-1-nLuc, SHC014-nLuc, SARS-CoV-2-nLuc, and SARS-CoV-2 on either HeLa-hACE2 or Vero target cells. SARS-CoV, WIV-1-nLuc, SHC014-nLuc, and SARS-CoV-2 nLuc assays were performed on Vero target cells. N.D., not determined; N.A., not applicable due to maximal neutralization plateau at <50% neutralization. (**D**) Authentic SARS-CoV-2 neutralization titrations performed using either HeLa-hACE2 (left) or Vero (right) target cells. The curves were fit by nonlinear regression. Error bars represent SD. All data are representative of at least two independent experiments.

Yeast display libraries were generated by introducing diversity into the heavy- and light-chain-variable genes of ADI-55688, ADI-55689, and ADI-56046 through oligonucleotide-based mutagenesis and transformation into *Saccharomyces cerevisiae* by homologous recombination (*8*). After four rounds of selection with a recombinant SARS-CoV-2 S1 protein, improved binding populations were sorted, and 20 to 50 unique clones from each lineage were screened for binding to SARS-CoV-2 S (*10*) (Fig. 1, A and B, and fig. S1). The highest-affinity binders from each of the three lineages bound to the S protein with monovalent equilibrium dissociation constants (K_D_s) in the picomolar range, representing 25- to 630-fold improvements in binding relative to their respective parental clones and surpassing the affinities of several clinical-stage nAbs (S309, REGN10987, REGN10933, and CB6/LY-CoV16) (Fig. 1B and fig. S2A) (*4*, *11*, *12*). To determine whether the improvements in SARS-CoV-2 S binding affinity translated to enhanced neutralization potency, we selected 9 to 14 affinity-matured progeny from each lineage and evaluated them for SARS-CoV-2 neutralizing activity in a murine leukemia virus (MLV) pseudovirus assay (*13*). All of the affinity-matured antibodies showed improved neutralizing activity relative to that of their parental clones, and the most-potent neutralizers from each lineage (ADG-1, ADG-2, and ADG-3) displayed neutralization half-maximal inhibitory concentrations (IC_50_s) that were comparable to or lower than those observed for the clinical SARS-CoV-2 nAb controls (Fig. 1B). Notably, however, we observed no correlation between the binding affinities and neutralization potencies of ADG-1, -2, and -3 and the clinical-stage antibodies, suggesting that neutralization potency is more tightly linked to fine epitope specificity than binding affinity to prefusion S (fig. S2B).

Because in vitro engineering can lead to polyreactivity with potential risks of off-target binding and accelerated clearance in vivo (*14*), we evaluated ADG-1, ADG-2, and ADG-3 in a polyreactivity assay that is predictive of serum half-life in humans (*15*). All three antibodies lacked polyreactivity in this assay, indicating a low risk for poor pharmacokinetic behavior (fig. S3). The three antibodies also showed low hydrophobicity, a low propensity for self-interaction, and thermal stabilities within the range observed for clinically approved antibodies (fig. S3). Thus, the process of in vitro engineering appeared not to negatively affect biophysical properties that are often linked to characteristics such as serum half-life, ease of manufacturing, ability to formulate to high concentrations, and long-term stability.

## Neutralization breadth and potency of down-selected antibodies

To determine whether the process of affinity engineering affected neutralization breadth, we evaluated ADG-1, ADG-2, and ADG-3, as well as their respective parental antibodies, for neutralizing activity against a panel of representative clade 1 sarbecoviruses (SARS-CoV, SHC014-nLuc, SARS-CoV-2-nLuc, and WIV-1-nLuc). SHC014 and WIV-1 were selected because these two bat SARS-like viruses readily replicate in primary human airway cells, suggesting the potential for direct transmission to humans (*16*, *17*). Consistent with the MLV–SARS-CoV-2 assay results, ADG-2 displayed highly potent neutralizing activity against authentic SARS-CoV-2-nLuc, with an IC_50_ comparable to or lower than those observed for clinical-stage SARS-CoV-2 nAbs (*4*, *11*, *12*, *18*) (Fig. 1C and fig. S4). Furthermore, in contrast to the benchmark nAbs, ADG-2 displayed high neutralization potency against authentic SARS-CoV and the two bat SARS-related viruses, with IC_50_s between 4 and 8 ng/ml (Fig. 1C and fig. S4). ADG-3 and the clinical nAb S309 also cross-neutralized all four sarbecoviruses but with markedly lower potency than that of ADG-2.

On the basis of its potent cross-neutralization and favorable biophysical properties, we selected ADG-2 as a lead therapeutic candidate and confirmed its potent neutralizing activity in an alternative authentic SARS-CoV-2 neutralization assay (IC_50_ ~ 1 ng/ml) (Fig. 1, C and D, and fig. S4). Because SARS-CoV-2 D614→G (D614G) has emerged as the dominant pandemic strain (*19*), we also performed neutralization studies with MLV–SARS-CoV-2 D614G and confirmed that ADG-2 retains potent neutralizing activity against this strain (fig. S5).

## ADG-2 displays broad binding activity to clade 1 sarbecovirus RBDs

**Fig. 2:**
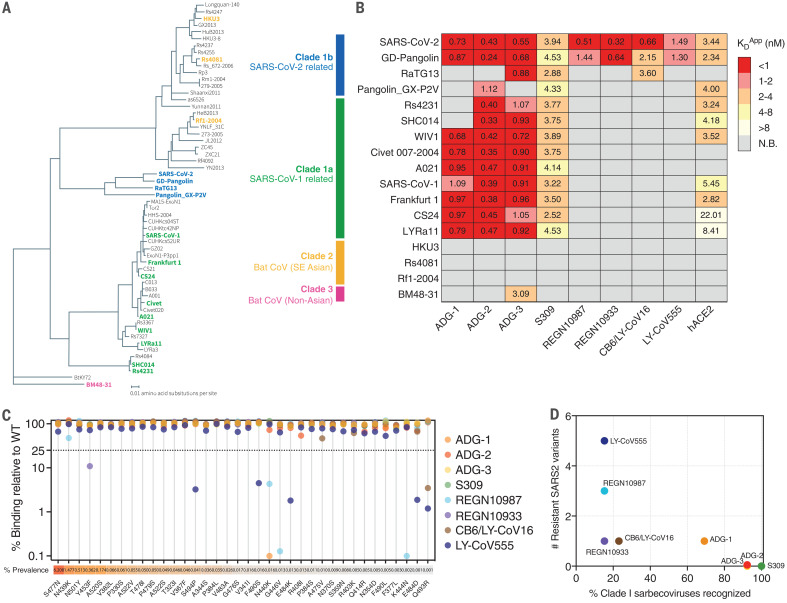
Breadth of antibody binding to diverse sarbecoviruses and circulating SARS-CoV-2 variants. (**A**) Phylogenetic tree of 57 sarbecoviruses constructed via MAFFT (Multiple Alignment using Fast Fourier Transform) and maximum likelihood analysis of RBD subdomain 1 amino acid sequences extracted from the European Nucleotide Archive and GISAID database. Representative sarbecovirus RBDs selected for further study are denoted in bold and colored according to their canonical phylogenetic lineages. (**B**) Heatmap of antibody and recombinant hACE2 binding to yeast-displayed RBDs from 17 representative sarbecoviruses, grouped by phylogenetic lineages. K_D_^App^ values were calculated by normalized nonlinear regression fitting. N.B., nonbinder under the conditions of this assay. (**C**) Antibody binding to naturally occurring SARS-CoV-2 RBD variants displayed on the surface of yeast. SARS-CoV-2 sequences were retrieved from the GISAID database on 14 July 2020 (*n* = 63,551 sequences). Antibody binding signal was normalized to RBD expression and calculated as percent binding of the variant relative to the WT SARS-CoV-2 RBD, assessed at their respective K_D_^App^ concentrations for the WT construct. The prevalence of each variant, calculated from deposited GSAID sequences on 9 December 2020 (*n* = 211,539 sequences), is shown as a percentage of the total number of sequences analyzed. (**D**) Correlation between the number of resistant SARS-CoV-2 variants and percentage of clade 1 sarbecovirus RBDs recognized. All data are representative of two independent experiments.

We further assessed the breadth of sarbecovirus recognition by ADG-2 by measuring its apparent binding affinity (K_D_^App^) to a panel of 17 representative sarbecovirus receptor binding domains (RBDs) expressed on the surface of yeast (*20*). Thirteen viruses were selected from clade 1—representing the closest known relatives of SARS-CoV-2 (GD-Pangolin and RaTG13) to the most divergent (SHC014 and Rs4231)—as well as four viruses from the distantly related clades 2 and 3, which do not use ACE2 as a host receptor (*21*) (Fig. 2A). Recombinant hACE2-Fc and the benchmark SARS-CoV-2 nAbs described above were included as controls. In agreement with prior reports (*10*, *20*), hACE2 recognized only clade 1 RBDs and bound with higher affinity to SARS-CoV-2 than to SARS-CoV (Fig. 2B).

Consistent with their broadly neutralizing activities, S309, ADG-2, and ADG-3 displayed broad binding reactivity to clade 1 sarbecovirus RBDs, with ADG-2 and ADG-3 strongly binding 12 of 13 viruses and S309 binding all 13 (Fig. 2B). Notably, ADG-2 bound with high affinity (K_D_^App^ = 0.24 to 1.12 nM) to every clade 1 sarbecovirus RBD that exhibited detectable hACE2 binding, supporting the high degree of ADG-2 epitope conservation among sarbecoviruses that can use hACE2 as a receptor. By contrast, ADG-1 bound to only 9 of 13 viruses, and CB6/LY-CoV16, LY-CoV555, REGN10987, and REGN10933 bound to only the closest evolutionary neighbor(s) of SARS-CoV-2, consistent with their narrow neutralization profiles (Figs. 2B and 1C).

Prior studies have shown that RBD mutants that are resistant to commonly elicited SARS-CoV-2 nAbs are circulating in the human population (*19*, *22*–*24*). We therefore sought to assess the breadth of ADG-2 binding to naturally circulating SARS-CoV-2 variants that contain single amino acid substitutions in the RBD. ADG-1, ADG-3, and the benchmark SARS-CoV-2 nAbs were included as comparators. Using the yeast surface display platform, we expressed the 30 most frequently observed SARS-CoV-2 RBD variants reported in the GISAID database as well as 6 naturally circulating SARS-CoV-2 variants reported to be resistant to certain SARS-CoV-2 nAbs (*19*, *22*, *25*). One or more of the 36 SARS-CoV-2 variants exhibited loss of binding to ADG-1, CB6/LY-CoV16, LY-CoV555, REGN10987, and REGN10933, as defined by >75% loss relative to the wild-type (WT) construct (Fig. 2C). Notably, the loss-of-binding variants identified for REGN10987 and REGN10933 partially overlapped with those identified in previous in vitro neutralization escape studies, validating the use of RBD display for the prediction of antibody escape mutations (*26*). By contrast, ADG-2, ADG-3, and S309 bound to all 36 variants at levels ≥50% of those for WT SARS-CoV-2 (Fig. 2C). This result, combined with the substantial neutralization breadth observed for these three mAbs (Figs. 1C and 2, B and D), indicates a potential link between epitope conservation and resistance to viral escape. Finally, all of the antibodies retained high-affinity binding to the N501Y variant, which is the only RBD mutation present in the rapidly spreading B.1.1.7 lineage (*27*), suggesting that this newly emerging SARS-CoV-2 strain is likely susceptible to neutralization by these antibodies.

## ADG-2 binds to a conserved neutralizing epitope overlapping the hACE2 binding site

**Fig. 3:**
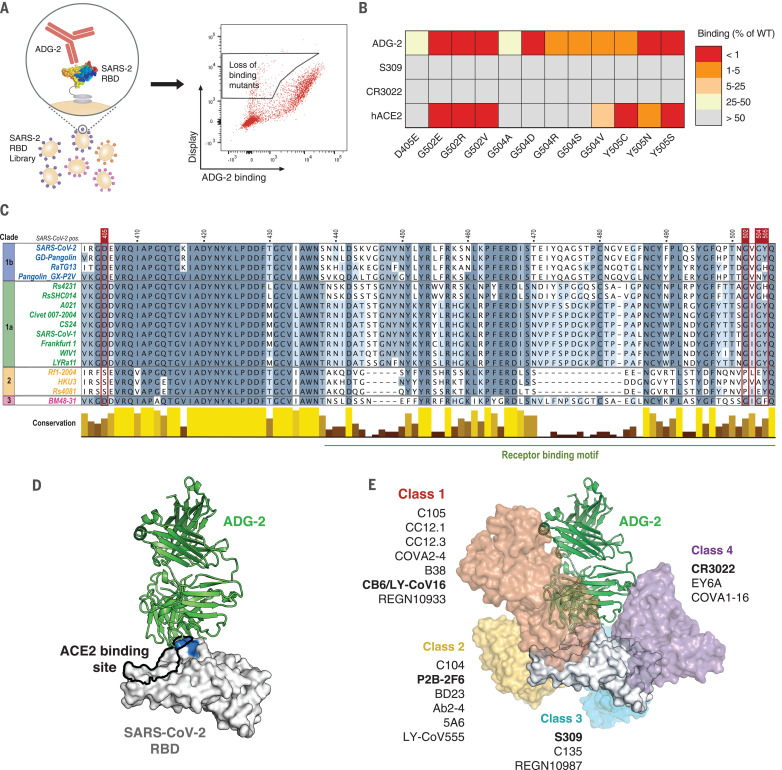
ADG-2 binds to a conserved RBD epitope overlapping the hACE2 binding site. (**A**) Schematic showing the generation and selection of a mutagenized, yeast surface–displayed SARS-CoV-2 RBD library to identify mutations that knock down ADG-2 binding. (**B**) Heatmap showing mutations that abrogate binding of ADG-2 to the SARS-CoV-2 RBD. S309 and CR3022, which bind nonoverlapping epitopes distinct from the ADG-2 binding site, are included to control for mutations that globally disrupt the conformation of the RBD. Values indicate percent antibody or recombinant hACE2-Fc binding to the mutant SARS-CoV-2 RBD relative to the WT SARS-CoV-2 RBD, assessed at their respective EC_80_ concentrations (80% effective concentrations) for the WT RBD construct. (**C**) Protein sequence alignment of representative sarbecovirus RBDs, with sequences colored by percentage sequence identity and conservation shown as a bar plot. Positions delineating the receptor binding motif are based on the SARS-CoV-2 RBD. Residues determined to be important for ADG-2 binding, on the basis of the data shown in (B), are denoted in red. Single-letter abbreviations for the amino acid residues are as follows: A, Ala; C, Cys; D, Asp; E, Glu; F, Phe; G, Gly; H, His; I, Ile; K, Lys; L, Leu; M, Met; N, Asn; P, Pro; Q, Gln; R, Arg; S, Ser; T, Thr; V, Val; W, Trp; and Y, Tyr. (**D**) Cryo-EM reconstruction of the SARS-CoV-2 RBD bound by ADG-2, with ADG-2 knockdown mutations (blue) and the hACE2 binding site (black outline) highlighted. (**E**) Structures of previously reported antibodies (bold), representing frequently observed SARS-CoV-2 nAb classes 1 to 4, are overlaid on the ADG-2 structure (D), with additional representative SARS-CoV-2 nAbs listed.

To gain further insight into the antigenic surface recognized by ADG-2, we generated a mutagenized yeast surface display RBD library and performed rounds of selection to identify RBD variants that exhibited loss of binding to ADG-2 relative to the WT construct (Fig. 3A and fig. S6, A and B). To exclude mutations that globally disrupt the conformation of the RBD, a final round of positive selection was performed using a mixture of recombinant hACE2-Fc and two RBD-directed mAbs (S309 and CR3022) that target nonoverlapping epitopes distinct from the ADG-2 binding site (*4*, *28*) (figs. S6B and S7). Selected RBD mutants were individually tested for binding to ADG-2, recombinant hACE2-Fc, CR3022, and S309 to confirm site-specific knockdown mutations (fig. S6C). Substitutions at only four RBD positions—D405E, G502E/R/V, G504A/D/R/S/V and Y505C/N/S—specifically abrogated ADG-2 binding (Fig. 3B). These four residues are highly conserved among the clade 1 sarbecovirus subgenus and invariant among SARS-CoV-1, SARS-CoV-2, SHC014, and WIV1 viruses (Fig. 3C), providing a molecular explanation for the breadth of binding and neutralization exhibited by ADG-2. Consistent with the conservation of these residues among clade 1 sarbecoviruses, none of the substitutions that affected ADG-2 binding were present in full-length SARS-CoV-2 sequences deposited in the GISAID database at a frequency >0.001% as of 9 December 2020. In addition, three of the four identified mutations that abrogate ADG-2 binding lie within the hACE2 binding site (*29*), and at least one mutation at each position (G502E/R/V, G504V, and Y505C/N/S) also abrogated hACE2 binding (Fig. 3B), likely accounting for their absence among circulating SARS-CoV-2 isolates. These results suggest that the evolutionary conservation of the ADG-2 epitope is likely directly linked to ACE2 binding.

To support the results of this experiment, we performed low-resolution cryo–electron microscopy (cryo-EM) of the complex of ADG-2 bound to prefusion-stabilized SARS-CoV-2 S. This yielded a ~6-Å resolution three-dimensional reconstruction that clearly had at least one ADG-2 Fab bound to an RBD in the up conformation and allowed us to dock in previously determined high-resolution models of the SARS-CoV-2 spike and a homologous Fab (Fig. 3D; fig. S8, A to D; and table S1). Consistent with our fine epitope mapping experiments (Fig. 3B and fig. S7C), the epitope recognized by ADG-2 overlaps the hACE2-binding site and each position identified by epitope mapping clustered to the cleft between the heavy and light chains of ADG-2 (Fig. 3D). This epitope also partially overlaps with those recognized by frequently observed “class 1” SARS-CoV-2 nAbs (*30*) (Fig. 3E). However, in contrast to previously reported nAbs in this class, ADG-2 binds with a divergent angle of approach and displays broadly neutralizing activity (*30*) (Figs. 3E and 1C and fig. S8E). Thus, ADG-2 binds to a highly conserved motif through a distinct angle of approach.

## ADG-2 potently triggers Fc-mediated effector functions in vitro

**Fig. 4:**
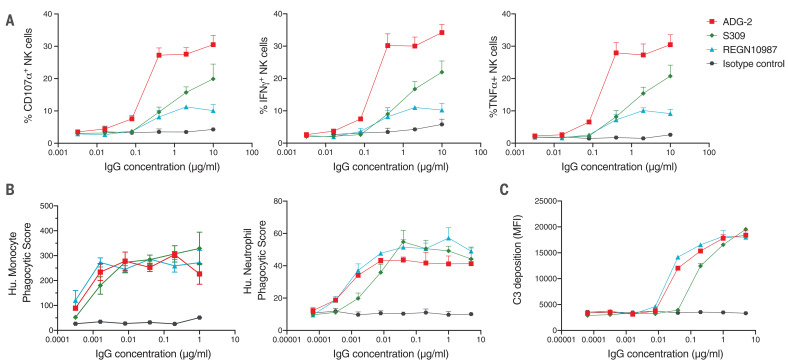
ADG-2 triggers Fc-mediated effector functions. The indicated antibodies were assessed for the ability to induce Fc-mediated effector functions against RBD-coated targets at varying concentrations. (**A**) Primary human NK cells were analyzed for surface expression of CD107a, indicating degranulation (left), and the production of interferon-γ (IFNγ) (middle) or tumor necrosis factor–α (TNFα) (right) after incubation with antibody-RBD immune complexes for 5 hours. IgG, immunoglobulin G. (**B**) Antibody-mediated phagocytosis of RBD-coated fluorescent beads by differentiated THP-1 monocytes (left) or HL-60 neutrophils (right) was measured after incubation with immune complexes for 18 hours. Hu., human. (**C**) Antibody-mediated complement deposition was measured by detection of complement component C3 onto RBD-coated fluorescent beads after incubation of guinea pig complement with immune complexes for 20 min. MFI, mean fluorescence intensity. Error bars represent SD. All data are representative of two independent experiments.

Because Fc-mediated effector functions can contribute to protection independently of viral neutralization, we assessed the ability of ADG-2 to induce antibody-dependent natural killer (NK) cell activation and degranulation, antibody-dependent cellular phagocytosis mediated by monocytes and neutrophils, and antibody-mediated complement deposition using in vitro effector function assays (*31*). Benchmark SARS-CoV-2 nAbs S309 and REGN10987 were included as comparators. Notably, though ADG-2, S309, and REGN10987 showed comparable recruitment of phagocytosis (Fig. 4B), these antibodies differed with respect to complement deposition and NK cell activation (Fig. 4, A and C): S309 showed reduced complement deposition compared with ADG-2 and REGN10987, and ADG-2 showed superior NK cell activation to both S309 and REGN10987 (Fig. 4). In summary, ADG-2 robustly triggers diverse Fc-mediated effector activities with potencies comparable or superior to those of current SARS-CoV-2 clinical antibodies.

## ADG-2 broadly protects in murine models of SARS and COVID-19

**Fig. 5:**
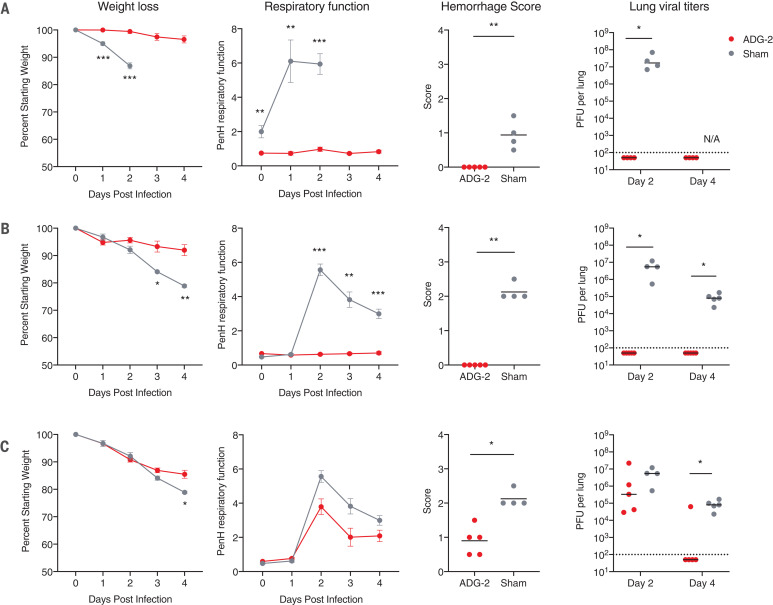
Prophylactic and therapeutic administration of ADG-2 protects mice from SARS-CoV– and SARS-CoV-2–associated disease. Efficacy of prophylactic treatment with ADG-2 in (**A**) SARS-CoV–MA15 and (**B**) SARS-CoV-2–MA10 challenge models. A single dose of ADG-2 or sham treatment was delivered intraperitoneally 12 hours before infection. Mouse body weight and respiratory function were monitored for 4 days. Gross lung hemorrhage scores were determined on day 2 (MA15) or day 4 (MA10) after infection, and lung viral titers were measured on days 2 and 4 after infection. (**C**) Therapeutic treatment with ADG-2 or sham treatment 12 hours after SARS-CoV-2–MA10 infection. Mouse body weight, respiratory function, gross hemorrhage scores (day 2), and lung viral titers (days 2 and 4) were assessed as described above. Statistical comparisons were made using Mann-Whitney *U* tests or two-sided *t* tests with Holm-Sidak corrections for multiple comparisons (*P < 0.05, **P < 0.01, ***P < 0.001). Dotted lines indicate the limit of detection. Horizontal bars indicate mean or geometric mean.

Finally, we tested the ability of ADG-2 to provide broad in vivo protection in immunocompetent mouse models of SARS and COVID-19 using mouse-adapted SARS-CoV (MA15) and SARS-CoV-2 (MA10), respectively (*32*, *33*). Balb/c mice were prophylactically treated with either 200 μg of ADG-2 or phosphate-buffered saline (PBS) via intraperitoneal injection 12 hours before intranasal challenge with an MA15 or MA10 dose of 10^3^ plaque-forming units (PFU). All mice were monitored daily for weight loss and changes in respiratory function, and groups of mice were euthanized at day 2 or 4 postinfection to allow for measurement of virus replication in the lung and analysis of lung histopathology. We observed substantial, progressive weight loss in sham-treated mice infected with both viruses along with increases in enhanced pause (Penh), a calculated measure of airway resistance (*33*). By contrast, mice treated prophylactically with ADG-2 demonstrated minimal weight loss, no change in Penh, and no signs of gross pathology at the time of harvest (Fig. 5, A and B). Furthermore, prophylactic antibody treatment prevented viral replication in the lungs at both 2 and 4 days postinfection (dpi). We next investigated the ability of ADG-2 to act antivirally against SARS-CoV-2 MA10 in a therapeutic setting. Mice were treated with 200 μg of ADG-2 or PBS 12 hours after intranasal challenge with a 10^3^-PFU dose of MA10. Mice given therapeutic ADG-2 had intermediate levels of weight loss, moderate respiratory function changes, and some gross lung pathology—significantly more than prophylactically treated mice but significantly less than sham-treated mice (Fig. 5C). Therapeutic antibody treatment also resulted in a significant reduction in lung viral loads at 4 dpi, but not at 2 dpi, relative to sham-treated mice. We conclude that ADG-2 treatment can reduce disease burden in mice infected with both SARS-CoV MA15 and SARS-CoV-2 MA10.

## Discussion

Since the beginning of the COVID-19 pandemic, a plethora of potently neutralizing SARS-CoV-2 antibodies have been isolated, and some have rapidly advanced to clinical trials (*34*). However, the epitopes recognized by most of these nAbs are highly variable among other clade 1 sarbecoviruses, hence limiting their neutralization breadth and increasing their susceptibility to antibody escape mutations (*22*). Here, we describe an engineered antibody that neutralizes SARS-CoV-2 with a potency that rivals current lead SARS-CoV-2 clinical nAbs but also broadly neutralizes other clade 1 sarbecoviruses, potently triggers Fc-mediated effector functions, and provides significant protection against SARS and COVID-19 in mouse models. Thus, ADG-2 represents a promising candidate for the prevention and treatment of not only COVID-19, but also future respiratory diseases caused by pre-emergent SARS-related CoVs. Furthermore, our fine epitope mapping and structural studies demonstrate that ADG-2 employs a distinct angle of approach to recognize a highly conserved epitope that overlaps the receptor binding site. This epitope represents an Achilles’ heel for clade 1 sarbecoviruses and hence is an attractive target for the rational design of “pan-SARS” vaccines that aim to elicit similar broadly protective antibodies.

## Supplementary Materials

science.sciencemag.org/content/371/6531/823/suppl/DC1 Materials and Methods Figs. S1 to S8 Tables S1 and S2 References (*35*–*50*) MDAR Reproducibility Checklist

## Appendix

View/request a protocol for this paper from *Bio-protocol*.
